# The quantum foundations of utility and value

**DOI:** 10.1098/rsta.2022.0286

**Published:** 2023-08-07

**Authors:** Cal R. Abel

**Affiliations:** Signal Power and Light, Inc, Cordova, AL, USA

**Keywords:** utility, value, game theory, entropy, quantum theory

## Abstract

This work presents a generalization of game theory and new perspectives on utility and value. Using quantum formalism, we prove that classical game theory is a special case of quantum game theory. We show that the von Neumann entropy and von Neumann–Morgenstern utility are equivalent and that the Hamiltonian operator represents value.

This article is part of the theme issue ‘Thermodynamics 2.0: Bridging the natural and social sciences (Part 1)’.

## 1. Introduction

The tricky part of integrating two seemingly unrelated scientific fields, quantum theory and economics, is finding the relevant analogies. The first and most obvious question is, what does quantum theory have to do with economics? Quantum theory provides the basis for the unobserved choice of an individual, representing the state of a game before a decision is made/acted upon.

The quantum world is a world in which observation has not yet occurred. Here, choice exists as simultaneous potentials of multiple possibilities in what is known as a quantum superposition. Then, making things more abstract, the system decoheres from its quantum state by becoming entangled with its environment, now behaving in a purely classical context.

Like in physics, the classical world is the only world we can measure/observe; the classical observed world is the rational world. Because our observations must obey classical probability theory, it becomes apparent that, in economics, decoherence is the act of making a decision, and that the quantum state represents unrealized choice.

Thus, classical game theory never truly represented the decision-making process, which is why there has been a considerable effort, particularly in mathematical psychology [1–4], to develop quantum decision theory in order to overcome classical theory’s shortcomings. Many of these authors have correctly identified that the classical-quantum divide rests upon making a decision. However, their development of quantum theory remains restrained.

Our most significant departure from classical [5–8] and quantum game theories [1–4,9,10] is the application of statistical ensembles. Ensembles are an entirely foreign economic concept; their exclusion is the single most significant impediment to economic theory.

Gibbs first introduced statistical ensembles in *Elementary Principles in Statistical Mechanics* [11]. His use of ensembles was so natural and powerful that von Neumann readily adapted them to quantum statistical mechanics [12] with *no* modification.

Ensembles are neither classical nor quantum structures; they are just a means of formally representing a system of one or of many things. Had Savage used ensembles, he would have resolved the Allais paradox [13] trivially. Ensembles show us that there is no single outcome for equilibria; there is a distribution of outcomes. One exception to this rule is a Nash equilibrium, which is a very, very special case. For a Nash equilibrium to exist, an ensemble must satisfy two conditions: (a) one individual’s choice does not prohibit another’s choice; choice is symmetric and (b) ensembles must have a very low activity (temperature).

Both of these conditions are the same as those for a Bose–Einstein condensate, and while they do exist in nature, they are infrequent. In every other circumstance, the ensemble will *always* be a distribution of outcomes.

Since an ensemble’s observable states are a probability distribution, Gibbs noted that they possess an index of probability ([11], p. 20), which would, 55 years later, be identified as Shannon’s information entropy [14,15]. Neither did Gibbs benefit from Jaynes’ universal law that entropy is always maximized, yet Gibbs intuitively used it.

Some economists have tried to relate entropy in economics to utility [16]. However, in every attempt, they did not understand that entropy came from ensembles and thus could not connect utility and entropy formally. To resolve this shortcoming, we equate the von Neumann–Morgenstern utility and the von Neumann entropy using Pfanzagl’s representation theory ([7], pp. 195–220).

Other than the von Neumann entropy, the other fundamental quantum operator is the Hamiltonian, $\hat{H}$. It first appears in the derivation of the Schrödinger equation (2.3), which is not the first time that either has appeared in an economics text [2,10]. However, it is the first time the Hamiltonian has been used to define the canonical economic distribution.

Using the maximum entropy approach, Gibbs was the first to derive the classical canonical distribution ([11], pp. 32–45). Applying the Gibbs ensemble, von Neumann derived the quantum version ([12], pp. 379–398). We derive the latter distribution for the Gibbs ensemble in economics,

1.1 $$\hat{\rho }=\frac{e^{-\beta \hat{H}}}{Z[\beta ]}.$$

Equation (1.1) represents the equilibrium distribution, $\hat{\rho }$, that occurs when the utility of a game is maximized. The term β is simply a Lagrangian multiplier of that maximization. The other term, Z[β], is a pure functional of β and not of the Hamiltonian.

Because the Hamiltonian defines the equilibrium distribution, it is apparent that the Hamiltonian represents a game’s value to the players. It can represent entangled players playing cooperative games, or it can represent independent players in non-cooperative games. Thus under a quantum framework, there is no distinction between these two games, and we see that the Hamiltonian represents value.

We are justified in calling our approach ‘microeconomics’ or even ‘macroeconomics’; these names do not acknowledge the formal application of statistical ensembles. Thus, as ensembles define statistical physics, they also define statistical economics.

## 2. Foundations of quantum game theory

Conceptually, the approach taken here is similar to Nash’s formalism [8]. The primary difference is that Nash uses real Euclidean spaces, and we use complex Hilbert spaces. Due to the similarity, we can directly compare Nash’s formalism with ours as we develop the axioms and recover Nash’s classical strategy vectors by passing their quantum counterparts to the classical limit.

The presented axioms restrict a game’s vector space and define what we can measure. They have three objectives: define a strategy vector’s structure, restrict measurements only to be real-valued and relate a strategy vector to probability theory. Once we have developed the axiomatic framework, we need to prove that any quantum observable possesses the three properties of what Pfanzagl calls the ‘safety equivalent wager’ ([7], p. 207). This ‘wager’ can represent any economic observable; Pfanzagl refers to these observables as ‘commodities’ associated with the payout of the wager ([7], p. 202).

Nash took the connection of the payout observable to vNM utility as being given [8]. Because we need to define utility’s observable formally, we cannot do the same. We use Pfanzagl’s utility functionals’ limited definition to identify the observable, a continuous increasing functional that is unique to a positive linear transformation and an additive constant ([7], pp. 208–209), to prove that the von Neumann entropy measures vNM utility.

### (a) Axioms of quantum game theory

#### (i) The space of games

The essential difference between quantum and classical theory is that the latter represents a strategy’s observed outcome; the former represents a strategy before making a decision. Where deciding collapses the wave function, causing a quantum strategy to behave classically.

We begin by defining the space of games.^1^

**Axiom 2.1.**

The space, G, of a game is represented by an n-dimensional complex Hilbert space. A pure strategy is a vector, |ψ⟩, where ⟨ψ|ψ⟩=1, that is a ray in G.^2^

We define a dual-vector space, $G^{\vee }$, composed of the vectors ⟨ψ| with a one-to-one correspondence between the vectors, |ψ⟩∈G and their Hermitian transpose, $\langle \psi |={|\psi \rangle }^{†}\in G^{\vee }$.

Like classical formalism, quantum formalism defines pure strategies but with some notable differences. The quantum formalism also adds a new vector, the basis vector. Basis strategy vectors, |j⟩, represent observed classical outcomes, are, by definition, orthogonal, and are equivalent to Nash’s pure strategies, $\pi_{j}$ ([8], p. 286).

A pure strategy is a superposition of basis strategies, $|\psi \rangle =\sum_{j}\alpha_{j}|j\rangle ,\;\alpha_{j}\in C$, that may or may not be entangled/communicative. Thus, pure quantum strategies represent a choice before it becomes a decision—a choice in an unobserved state—with probability amplitudes, $\alpha_{j}$.

#### (ii) Time evolution of isolated strategies

Economic observables possess a transformation that is orthogonal to the payout ([7], p. 208). Pfanzagl defines an observable X of a payout a∈A as possessing the relationship

2.1 $$X_{1}(a)≋\alpha X_{0}(a),$$

where ≋ means $X_{1}(a)-\alpha X_{0}(a)$ is independent of a, A is the set of payouts corresponding to independent events {P,Q,R,…}∈E, and E is the set of all possible events ([7], p. 208).

If we consider α as a function of time, it is apparent that it transforms the probability of an observable’s outcome from one time to another. This process is what a time-evolution operator, $\hat{U}(t)$, does when operating on a strategy vector, |ψ⟩ (0), which we adopt as Axiom 2.2.

**Axiom 2.2.**

The time-evolution of an isolated strategy is given by the unitary operator $\hat{U}(t)$ such that

$$|\psi \rangle \;(t)=\hat{U}(t)|\psi \rangle \;(0).^{3}$$

There is a more profound significance to our interpretation of Pfanzagl’s α–transformation; its unitary property is related to the conservation of probability ([18], p. 64).

#### (iii) The Hamiltonian

Pfanzagl left the α-transformation undeveloped, missing an essential observable associated with it, the Hamiltonian. We define the Hamiltonian operator, $\hat{H}$, through the time-dependent Schrödinger equation with a constant c determining the Hamiltonian’s units.

**Theorem 2.3. (Time-dependent Schrödinger equation)**

2.2 $$i\;c\frac{d}{dt}|\psi \rangle \;(t)=\hat{H}|\psi \rangle \;(t).$$

The Schrödinger equation only applies to isolated systems, where knowledge of the internal state has not been communicated to the surroundings. What is surprising is that these internal preferences are not stable over time with experimental evidence supporting equation (2.2) ([4], p. 769). Preference varies as a function of the Hamiltonian. It is for this reason that we consider the Hamiltonian as representing value. What drives an individual to consider different choices or sets of choices if it is not the evaluation of the relative value for each choice?

#### (iv) The basis of a game and its observables

We define an observable (a measurable quantity) as being,

**Axiom 2.4.**

An observable, $\hat{M}$, is a self-adjoint, Hermitian, operator that through the spectral theorem can be represented as $\hat{M}=\sum_{j}m_{j}{\hat{P}}_{j}$.

Because $\hat{M}$ is Hermitian, the eigenvalues, $m_{j}$, are real-valued and the projection operators ${\hat{P}}_{j}=|j\rangle \langle j|$ are orthogonal as are the eigenkets, |j⟩. The eigenkets form the orthonormal basis for G, with their number, J, representing the game’s dimensionality. Their orthogonality is equivalent to the Pfanzagl’s independence axiom ([7], p. 210).

To satisfy Pfanzagl’s requirement that the observables, ‘commodities’, be real-valued,

**Axiom 2.5.**

The possible outcomes of the observable $\hat{M}$ are its eigenvalues, $m_{j}$.

#### (v) Rationality

The rationality of economic actors played a significant role during EU’e development, [5–7], and its critiques, e.g. [13]. Rationality’s importance is unchanged.

To say something is rational, we must be able to measure/count what is being observed by Axiom 2.4. Thus, if it is observed, it is rational. Whether or not we can attribute a justification to a measurement is irrelevant to its rationality. Thus,

**Definition 2.6. (Economic rationality)**

Rational economic behaviour is observable behaviour.

#### (vi) Mixed states and expectations

Because |ψ⟩ is a unit vector and $\forall \;(|j\rangle ,|k\rangle )\in G,\;\langle k|j\rangle =\delta_{k,j}$,

2.3 $$\begin{array}{rl} 1 & \;=\langle \psi |\psi \rangle =\sum_{k,j}\langle k|\alpha_{k}^{†}\alpha_{j}|j\rangle =\sum_{k,j}\alpha_{k}^{†}\alpha_{j}\langle k|j\rangle =\sum_{k,j}\alpha_{k}^{†}\alpha_{j}\delta_{k,j}\\ \mathrm{and}\;\;1 & \;=\sum_{j}\alpha_{j}^{†}\alpha_{j}. \end{array}\}$$

Using the Born rule, the probability of outcome $m_{j}$ of observable $\hat{M}$ is

**Axiom 2.7. (Born rule)**

$$P(M=m_{j})=p_{j}=\alpha_{j}^{†}\alpha_{j}.$$

From equation (2.3), $\sum_{j}p_{j}=1$.

From the Born rule, any observable $\hat{M}$ that shares its basis with |ψ⟩ has the expectation,

$$\langle M\rangle =\langle \psi |\hat{M}|\psi \rangle =\sum_{k,j}\langle k|\alpha_{k}^{†}m_{j}|j\rangle \langle j|\alpha_{j}|j\rangle =\sum_{j}p_{j}m_{j}.$$

To describe a mixture of strategies, we need to be able to decompose the game into a set of sub-games.

**Axiom 2.8.**

The decomposition of a game into sub-games is the tensor product of its sub-games, $G_{12}=G_{1}⊗G_{2}$.

The strategies of a composite game may be entangled. In this case, a composite pure strategy of two games $|\psi_{A}\rangle =\sum_{j}\alpha_{j}|j\rangle$ and $|\psi_{B}\rangle =\sum_{k}\alpha_{k}|k\rangle$ would be,

$$|\psi_{AB}\rangle =\sum_{j\in J}\sum_{k\in K}\alpha_{j,k}|j\rangle ⊗|k\rangle =\sum_{j\in J}\sum_{k\in K}\alpha_{j,k}|j\rangle \langle k|.$$

If the sub-games are not entangled, they are separable, and $\alpha_{j,k}=\alpha_{j}\alpha_{k}$.

Axiom 2.8 does not allow us to represent a mixed strategy with a ket-vector. To resolve this, we take a similar approach to Nash’s mixed strategy vectors ([8], p. 286)) to define the density operator composed of a mixture of m pure strategies ([12], p. 296).

**Definition 2.9. (Mixed strategy)**

$$\hat{\rho }=\sum_{m}w_{m}|\psi_{m}\rangle \langle \psi_{m}|,\;w_{m}\in [0,1]\;\text{and}\;\sum_{m}w_{m}=1.$$

Using the trace operation, tr, the expectation of a mixed strategy observable is

$$\langle M\rangle =\sum_{m}w_{m}\langle \psi_{m}|\hat{M}|\psi_{m}\rangle =\mathrm{tr}\;[\hat{\rho }\hat{M}].$$

#### (vii) The quantum commutator

The presented formalism depicts strategies that change with time and observables that do not change; this is called the Schrödinger picture. We can also consider the equivalent Heisenberg picture, where the observations evolve with time, but the strategies do not. To arrive at the Heisenberg picture, we examine the operator $\hat{M}$,

2.4 $$\begin{array}{rl} \frac{d}{dt}\langle \hat{M}\rangle \;(t) & \;=\left\langle \frac{d\psi (t)}{dt}|\hat{M}|\psi (t)\right|+\left\langle \psi (t)|\hat{M}|\frac{d\psi (t)}{dt}\right\rangle \\ & \;=\frac{i}{c}\left\langle \psi (t)|\hat{H}\hat{M}|\psi (t)\right\rangle -\frac{i}{c}\langle \psi (t)|\hat{M}\hat{H}|\psi (t)\rangle \\ & \;=\frac{i}{c}\langle \psi (t)|\hat{H}\hat{M}-\hat{M}\hat{H}|\psi (t)\rangle \\ & \;=\frac{i}{c}\langle \psi (t)|[\hat{H},\hat{M}]|\psi (t)\rangle \end{array}$$

2.5 $$\begin{array}{rl} ic\frac{d}{dt}\langle \hat{M}\rangle \;(t) & \;=\sum_{k}\sum_{j}\langle k|\alpha_{k}^{*}(t)[\hat{M},\hat{H}]\alpha_{j}(t)|j\rangle \text{. Thus}\\ ic\frac{d}{dt}\hat{M} & \;=[\hat{M},\hat{H}]. \end{array}$$

Equation (2.5) is called the commutator.

Looking at the time evolution of $\hat{H}$, we find

2.6 $$\frac{d}{dt}\hat{H}=0.$$

Anything that commutes with the Hamiltonian, including itself, is conserved.

In the case where the Hamiltonian is not conserved, the solution is to break the Hamiltonian up into small enough time steps such that for each interval, the Hamiltonian is constant with time ([12], p. 351). The degree of the Hamiltonian’s discretization has fundamental limitations, which we develop in the next section.

Commuting Hermitian operators also possess a valuable property: the existence of (at least) one shared orthonormal basis.

**Lemma 2.10. (Simultaneous diagonalization)**

*If* $\hat{A}$ *and* $\hat{B}$ *are two commuting Hermitian operators, then there exists (at least) a common orthonormal basis*.

#### (viii) The uncertainty principle

We can express the uncertainty of any two simultaneous measurements as being

2.7 $$\Delta A\Delta B\geq \frac{1}{2}\left|\left\langle \left[\hat{A},\hat{B}\right]\right\rangle \right|.$$

Thus, if the operators commute, they are measurable simultaneously. If they do not commute, there is an absolute limit to the accuracy of simultaneous measurement, representing the degree to which they do not commute.

For example, consider the relationship between time and the Hamiltonian. Using equation (2.5) in equation (2.7), we find

2.8 $$\begin{array}{rl} & \Delta M\Delta H\geq \frac{1}{2}\left|ic\frac{d}{dt}\langle \hat{M}\rangle \right|\\ \mathrm{and}\;\; & \Delta t\Delta H\geq \frac{c}{2}. \end{array}\}$$

Equation (2.8) provides us with the minimum uncertainty for the simultaneous measurements of time, Δt, and the Hamiltonian, ΔH.

#### (ix) The classical limit

When we observe a particular event, the wave function, |ψ⟩, ‘collapses’ into one of its basis strategies with some observed probability, thus passing to the classical limit.

**Theorem 2.11.**

*Classical mixed strategy vectors*, $s=\sum_{j}c_{j}\pi_{j}$ *with* $1=\sum_{j}c_{j}$, *are equivalent to quantum mixed strategies at the classical limit*.

### (b) Foundations of utility

To resolve utility’s apparent disconnection from quantum formalism, we will use Pfanzagl’s clear and precise exposition of classical utility. Aside from Pfanzagl’s formal treatment of utility as a functional, his most significant contributions are: the three conditions that qualify observables for representation theory, each observable’s connection to utility, that representation theory contains the von Neumann–Morgenstern utility and that vNM utility is equivalent to his utility functional.

Our integration with Pfanzagl’s approach is in four areas: integrate his notation with ours, show that any quantum observable is in representation theory, represent his utility functional with a quantum observable and identify von Neumann entropy as utility’s quantum observable.

#### (i) Definitions

Before beginning, we need to define the operators and relations that Pfanzagl used in representation theory.

**Definition 2.12. (Events, outcomes and wagers)**

The set of all possible events, E, and the set of possible outcomes, A, can be used to express a simple wager, aPb, for a,b∈A and P∈E. Where outcome a occurs if event P obtains and outcome b occurs if P does not obtain ([7], p. 202).

**Definition 2.13. (Preference relations)**

Between any two possible outcomes of a particular event, their transitive relationship is either strict preference or indifference, ≻ or ∼, respectively. The preference relationship represents an inequality in utility, a≻b implies U(a)>U(b), while indifference represents an equality. When comparing wagers, these relationships also hold, e.g. aPb≿aQb ([7], pp. 202–203).

**Definition 2.14. (Safety equivalent wager)**

The operator, $\circ_{P}:\;a,b\in A\mapsto a\circ_{P}b\in A$, is defined by $aPb∼(a\circ_{P}b)P(a\circ_{P}b)$. The safety equivalent wager is $a\circ_{P}b$. ([7], p. 204).

**Definition 2.15. (Independence relationship)**

Let $U_{0},\;U_{1}:A\to R$. The relationship $U_{0}≋U_{1}$ means that $U_{0}(a)-U_{1}(a)$ is independent of a∈A ([7], p. 208).

#### (ii) The representation of quantum games

To show that the axioms of quantum theory satisfy Pfanzagl’s representation theory, we need to prove that any quantum observable’s expectation satisfies the safety equivalent wager’s properties and two additional conditions ([7], p. 207) taken as theorem 2.17.

**Theorem 2.16.**

(a) *Any quantum observable’s expectation satisfies the safety equivalent wager’s properties*:
  (i) $\circ_{P}$ *is intern except* P∼O *or* P∼E, *where* O *and* E *are the impossible and sure thing events*.
  (ii) $a\circ_{E}b=a$.
  (iii) $\circ_{P}$ *is increasing in both variables except* P∼O or P∼E.
  (iv) $(a,b)\to a\circ_{P}b$ *is continuous*.
  (v) $a\circ_{P}b=b\circ_{\bar{P}}a$ *for all* a,b∈A,P∈E.
  (vi) *The following statements are equivalent*:
    — $a\circ_{P}b∼a\circ_{Q}b$ *for at least one pair* a,b∈A.
    — $a\circ_{P}b∼a\circ_{Q}b$ *for all* a,b∈A.
    — P∼Q.
(b) *Indifference between observables requires that the operators commute.*

**Theorem 2.17.**

*All quantum observables are compatible with representation theory because they satisfy theorem 2.16 and the following conditions*:

$$\begin{array}{rlr} \text{(i) } & \; & \left(a\circ_{Q|P}b)\circ_{P}((c\circ_{Q|\bar{P}}d))=(a\circ_{P|Q}c)\circ_{Q}((b\circ_{P|\bar{Q}}d)\right)\\ \text{(ii ) } & \; & (a\circ_{Q|P}b)\circ_{P}b=a\circ_{PQ}b. \end{array}$$

#### (iii) The quantum utility observable

Pfanzagl proved that definition 2.18 is equivalent to vNM utility ([7], pp. 217–218). Using Pfanzagl’s approach, we will prove the existence of a quantum utility operator compatible with representation theory and equivalent to vNM utility.

**Definition 2.18. (The utility functional)**

There exist increasing and continuous functions U and $U_{R}(R\in E^{\prime })$ such that

(a) $U(a\circ_{P}b)≋U_{P}(a)+U_{\bar{P}}(b)$ and
(b) $U_{P}(a\circ_{Q|P}b)≋U_{PQ}(a)+U_{P\bar{Q}}(b)$.

The functions U and $U_{R}$ are unique up to linear transformations. If one of the functions, e.g. U, is fixed, all other functions are uniquely determined up to an additive constant. Moreover, $P∼P^{\prime }$ implies $U_{P}≋U_{P^{\prime }}$. Defining $U_{P}$ by U for P∼E and $U_{P}$ by zero for P∼O, the relation (a) holds for all P∈E and the relation (b) holds for all P,Q∈E with P≁O ([7], pp. 208–209).

Defining s(P) as the subjective probability functional of event P∈E and U(a) as the utility functional of payout a∈A, we assert

**Theorem 2.19.**

*If a representation*

$$U(\langle M\rangle )=s(\lambda_{1})U(m_{1})+(1-s(\lambda_{1}))U(m_{2}),$$

*exists and if* U *is increasing, then* $s(\lambda_{j})=\lambda_{j}$.

We note that the utility is a functional of an observable’s eigenvalues. This relationship makes utility an observable, denoted as $\hat{S}$, sharing the same basis of the original observable, |j⟩,

$$\hat{S}=\sum_{j}U(m_{j})|j\rangle \langle j|=\sum_{j}u_{j}|j\rangle \langle j|.$$

### (c) Philosophical foundations

We must explore two philosophical concepts used to justify two postulates necessary for creating economic ensembles. To do this, we turn to political philosophy adopting two principles similar to Rawls’ ‘veil of ignorance’ and the ‘first principle of justice’ [19]. Since Rawls does not explicitly develop the indistinguishability and independence principles, we need to deduce them from generalizations of the veil of ignorance and the first principle of justice, respectively.

#### (i) Indistinguishability principle

We use Rawls’ veil of ignorance for guidance in developing the indistinguishability principle. ‘[The veil of ignorance] ensures that no one is advantaged or disadvantaged in the choice of principles by the outcome of natural chance or the contingency of social circumstances’ ([19], p. 12). The veil theorizes that we can create a set of fair rules by seeing ourselves in each other—the rules are ignorant of to whom they apply so that they will be acceptable to all. Observing society from within and being constrained by such a set of rules, we could not distinguish how the rules limit each other. Thus, the rules act as a uniform set of constraints.

Generalizing this concept, we see that uniformly constrained people would act similarly under similar circumstances and natural chance. We can extend this idea across time and see that given the same historical circumstance that we would have acted similarly to our ancestors, and they would act as we do now. This concept is why Justice is blind and why Common Law treats all case history as informing our decisions today. For these reasons, we adopt the principle that,

**Postulate 2.20. (Indistinguishability principle)**

Individuals are indistinguishable from one another.

#### (ii) Independence principle

The second philosophical point is that individuals act independently. We apply Rawls’ first principle of justice, ‘Each person is to have an equal right to the most extensive total system of equal basic liberties compatible with a similar system of liberties for all’ ([19], p. 302). To act under this principle, each person must act without impairing the action of another.

Similarly, the rules of society are comported so that society does not inhibit the actions of individuals and protects individuals from being inhibited by others. Thus,

**Postulate 2.21. (Independence principle)**

Ensembles of strategies are separable.

### (d) Permutations and symmetries

The indistinguishability principle has some unique consequences in quantum theory. The primary consequence is that strategies are isomorphic between people, meaning that we cannot, even in principle, distinguish between the members of a system.

Take a system of two indistinguishable individuals, Alice and Bob, with strategy vectors $|\psi_{A}\rangle =\sum_{j}\alpha_{j}|j\rangle$ and $|\psi_{B}\rangle =\sum_{k}\alpha_{k}|k\rangle$, respectively, with |j⟩=|k⟩ and $\alpha_{j}=\alpha_{k},\;\forall j=k$. Their combined strategy vectors is $|\psi_{A},\psi_{B}\rangle =\sum_{j,k}\alpha_{j,k}|j\rangle ⊗|k\rangle$.

To describe other possible configurations, we use the permutation operator, $\hat{P}$,

$$|\psi_{A},\psi_{B}\rangle =\hat{P}|\psi_{B},\psi_{A}\rangle .$$

Because Alice and Bob are indistinguishable, $\hat{P}$ has eigenvalues of +1 and −1, being either symmetric or antisymmetric, respectively. Alice and Bob can share the same possible outcome in the symmetric case. However, in the antisymmetric case, attaining a particular outcome for one individual precludes the same outcome for the other.

By observing the quantum system as a participant or as an outside observer, the quantum strategies decohere, resulting in their collapse. The problem is that we could not tell who Alice or Bob was, just that one had a particular outcome, and the other had another. After the strategies collapse, we could label each strategy based on the observed outcome and then track the outcomes classically; however, such identification is a *post hoc* classical construction. The classical view allows us to track an individual’s history, a quantum impossibility.

### (e) Economic ensembles and their entropies

We can now formally develop economic ensembles, the mathematical representations of populations. Gibbs described the basic formulation of an ensemble as being ‘… a great number of independent systems, identical in nature, but differing in phase, i.e. in their condition with respect to [state/strategy] ([11], p. 5).’ von Neumann referred to this ensemble as the Gibbs ensemble ([12], p. 360), and we shall do the same.

#### (i) The von Neumann entropy

We now consider another operator central in constructing ensembles, the von Neumann entropy ([12], p. 380),

**Definition 2.22. (von Neumann entropy)**

$$S(\hat{\rho })=-\mathrm{tr}\;[\hat{\rho }\log\hat{\rho }].$$

Entropy shares the density matrix’s orthonormal basis. The distinction between the two are their eigenvalues: $\hat{\rho }|j\rangle =\lambda_{j}|j\rangle$ and $\log\hat{\rho }|j\rangle =\log\lambda_{j}|j\rangle$ ([12], p. 379).

Entropy is essential because it represents the multiplicity of system configurations. As Boltzmann pointed out, entropy is the ensemble’s log-multiplicity, S=log⁡Ω. The multiplicity is always defined, whether the system is classical or not.

In the quantum context, pure strategies have a von Neumann entropy of 0. The degree to which an ensemble is entangled results in a reduction of the von Neumann entropy from the ensemble’s Shannon entropy. The decomposition of a density matrix demonstrates this effect. The Shannon entropy of the strategy weights is $H(\text{p})=-\sum_{m}p_{m}\log p_{m}$ with the elements of the probability vector, p, defined as $\hat{\rho }=\sum_{m}p_{m}|\psi_{m}\rangle \langle \psi_{m}|,$ where $|\psi_{m}\rangle$ are pure strategies, and compare that with the von Neumann entropy,^4^

$$S(\hat{\rho })\leq H(\text{p}),$$

where the condition of equality only holds when the pure strategies are orthogonal ([20], pp. 101).

There are several important properties of von Neumann entropy:

— The entropy of a Gibbs ensemble is first-order homogeneous ([12], p. 379) and ([20], p. 108)
  $$S({\hat{\rho }}_{1}⊗{\hat{\rho }}_{2})=S({\hat{\rho }}_{1})+S({\hat{\rho }}_{2})=2S({\hat{\rho }}_{1}),\;{\hat{\rho }}_{1}={\hat{\rho }}_{2}.$$
— Entropy is a concave functional ([20], p. 102). For α∈[0,1],
  $$\begin{array}{rl} & \alpha S({\hat{\rho }}_{1})+(1-\alpha )S({\hat{\rho }}_{2})\leq S(\alpha {\hat{\rho }}_{1}+(1-\alpha )x{\hat{\rho }}_{2})\\ & \;\leq \alpha S({\hat{\rho }}_{1})+(1-\alpha )S({\hat{\rho }}_{2})+H(\alpha ,1-\alpha ). \end{array}$$
— Entropy is continuous when $\mathrm{tr}\;[{\text{e}}^{-\beta \hat{H}}]<\infty$ ([20], p. 104).
— Entropy is unique to an additive constant, probability’s normalization factor ([21], p. 92).
— Entropy is subadditive and constrained by the Araki–Lieb triangle inequality ([20], p. 104) and ([20], p. 109),
  $$|S({\hat{\rho }}_{1})-S({\hat{\rho }}_{2})|\leq S({\hat{\rho }}_{12})\leq S({\hat{\rho }}_{1})+S({\hat{\rho }}_{2}).$$

#### (ii) The equilibrium condition

Ensembles do not necessarily need to be in equilibrium. They are, after all, a generic construction with no restriction on their time evolution. However, by placing ensembles in an equilibrium state, we can derive several useful properties by maximizing their von Neumann entropy.

Conversely, one can adopt the formalism of non-equilibrium statistical mechanics and relax this constraint, which is a well-defined process. However, we will only consider the equilibrium condition here. We define an ensemble in statistical equilibrium as

**Definition 2.23. (Statistical equilibrium)**

$$\frac{i}{c}\frac{d\hat{\rho }}{dt}=0.$$

We previously noted that strategies indifferent across time must commute with the Hamiltonian. Moreover, considering the uncertainty principle, the density matrix is also complementary with time. The consequence is that simultaneous measurement of preference and time is impossible, and there is an absolute minimum uncertainty, c/2. Said another way, if we want to know the equilibrium condition, we cannot know when it was, other than it was/is/could be.

#### (iii) The Gibbs ensemble

There are many ways we can develop the functional relationships of the Gibbs ensemble. The most notable derivations of these relationships come from Gibbs ([11], pp. 33–45), von Neumann ([12], pp. 390–398), Jaynes [15] and Matsoukas ([21], pp. 88–97).^5^ All four derivations are extensions of their predecessors’ work, with Gibbs being the foundation. Every approach assumes the independence and indistinguishability principles when defining the ensemble.

Additionally, Gibbs and Jaynes assume that the microstates are separable—having equal *a priori* probability. von Neumann and Matsoukas do not make the same assumption, handling the situation differently but with equivalent results. We follow von Neumann’s approach and arrive at Matsoukas’ classical method. To illustrate the similarities, we will use Matsoukas’ notation.

Since we are talking about the observable outcomes of an ensemble, here is a list of what we know:

(i) Probability, $\hat{\rho }|j\rangle =\lambda_{j}|j\rangle ,\;\sum_{j}\lambda_{j}=1$.
(ii) Entropy, $\log\hat{\rho }|j\rangle =\log\lambda_{j}|j\rangle ,\;S(\hat{\rho })=\mathrm{tr}\;[\hat{\rho }\log\hat{\rho }]=\bar{s}$, what we are maximizing.
(iii) Number of people (size of the ensemble), $\hat{N}|j\rangle =n_{j}|j\rangle ,\;\sum_{j}n_{j}=N$.
(iv) Other observables, ${\hat{X}}_{k}|j\rangle =x_{k,j}|j\rangle ,\;\langle X_{k}\rangle =\mathrm{tr}\;[\hat{\rho }{\hat{X}}_{k}]={\bar{x}}_{k}$.^6^

We maximize entropy using the method of Lagrangian multipliers, $\beta_{k}$, with our observables

2.9 $$\hat{\rho }=\frac{1}{q}\;e^{-\sum_{k}\beta_{k}{\hat{X}}_{k}}.$$

The von Neumann entropy of equation (2.9) is

2.10 $$\bar{s}[{\bar{x}}_{1},\ldots ,{\bar{x}}_{K}]=\log q+\sum_{k}\beta_{k}{\bar{x}}_{k}.$$

The partition function, q, is the normalization constraint of probability;

**Definition 2.24. (Partition function)**

$$q[\beta_{1},\ldots ,\beta_{K}]=\mathrm{tr}\;\left[e^{-\sum_{k}\beta_{k}{\hat{X}}_{k}}\right].$$

For an ensemble total, its annotation for a given observable is the operator’s upper-case letter, e.g. $X_{k}\equiv N\;{\bar{x}}_{k}$. The partition function scales similarly, log⁡Q≡Nlog⁡q.

The ensemble’s total extensive entropy functional is

2.11 $$S[X_{1},\ldots ,X_{K},N]=N\;\bar{s}=(\log q)N+N\sum_{k}\beta_{k}{\bar{x}}_{k}=(\log q)N+\sum_{k}\beta_{k}X_{k}.$$

Because total entropy, equation (2.11) is first-order homogeneous, by Euler’s theorem, the Lagrangian multipliers are

$$\beta_{k}={\left(\frac{\partial S}{\partial X_{k}}\right)}_{X_{j\neq k},N}={\left(\frac{\partial \bar{s}}{\partial {\bar{x}}_{k}}\right)}_{{\bar{x}}_{j\neq k}}$$

and

$$\log q={\left(\frac{\partial S}{\partial N}\right)}_{X_{k}}.$$

Additionally, the $X_{k}$ are first-order homogeneous, while the $\beta_{k}$ are zeroth-order homogeneous.

The first-order homogeneous observables are called extensive, and the zeroth-order ones are called intensive. Thus, equation (2.10) expresses an intensive relationship because the relationship between $\bar{s}$ and the ${\bar{x}}_{k}$ is homogeneous to order 0. The differential of $\bar{s}$ is

2.12 $$d\bar{s}=\sum_{k}\beta_{k}d{\bar{x}}_{k}.$$

Exploring the intensive entropy functional further, we can express log⁡q in terms of $\bar{s}$ and the other variables as

$$\log q=\bar{s}-\sum_{k}\beta_{k}{\bar{x}}_{k}=\bar{s}-\sum_{k}\frac{\partial \bar{s}}{\partial {\bar{x}}_{k}}{\bar{x}}_{k}.$$

This representation shows that log⁡q is the Legendre transform of $\bar{s}$ and has the differential of,

2.13 $$d\log q=-\sum_{k}{\bar{x}}_{k}\;d\beta_{k}.$$

Thus,

$${\bar{x}}_{k}=-{\left(\frac{\partial \log q}{\partial \beta_{k}}\right)}_{\beta_{j\neq k}}.$$

#### (iv) The economic observables

Until now, we refrained from formally defining any economic observables other than connecting Nash’s mixed strategy vector to the density matrix, $\hat{\rho }$. In the development of quantum game theory, two observables need to be connected with economic concepts: the von Neumann entropy and the Hamiltonian.

But first, we define the quantity of money, the ‘payout’ which is a commodity, as the money observable, $\hat{M}$.^7^ The other commodities will continue using the ${\hat{X}}_{k}$ observable. These are straightforward definitions and should not be controversial.

Pfanzagl identified six conditions that a utility functional needs to satisfy in definition 2.18:

**Theorem 2.25. (Utility’s observable)**

*The von Neumann entropy measures von Neumann–Morgenstern utility*.

When we consider von Mises’ formulation of human action, it is that it is something ‘put into operation and transformed into agency’ ([22], p. 11). If, as people, we are defined by our actions and not our intent, then each time we act, we are changed in some manner. In physics, we describe the evolution of a physical system over a period of time as action, and it has dimensions of time × energy; it is closely related to the uncertainty principle. Because the physical operator for energy is the Hamiltonian, it is thus natural to attribute the economic Hamiltonian as part of human action.

What economic quantity does the Hamiltonian represent? We answer this with what we anticipate as being the most controversial definition,

**Definition 2.26. (Value)**

The Hamiltonian operator represents economic value.

We exchange money, goods and services because they have value. We often conflate value with utility, but this is for a good reason, as they are both increasing functionals of each other

2.14 $$\begin{array}{rl} & S[E,M,X_{k},N]=\left(\frac{1}{T}\right)E+\left(\frac{P}{T}\right)M+\left(\frac{-\mu }{T}\right)N+\sum_{k}\left(\frac{p_{k}}{T}\right)X_{k},\\ & E[S,M,X_{k},N]=T\;S-P\;M+\mu \;N-\sum_{k}p_{k}\;X_{k},\\ & dS[E,M,X_{k},N]=\left(\frac{1}{T}\right)dE+\left(\frac{P}{T}\right)dM+\left(\frac{-\mu }{T}\right)\;dN+\sum_{k}\left(\frac{p_{k}}{T}\right)dX_{k}\\ \mathrm{and}\;\; & dE[S,M,X_{k},N]=T\;dS-P\;dM+\mu \;dN-\sum_{k}p_{k}\;dX_{k}. \end{array}\}$$

Our notation will be the following for representing the Hamiltonian as economic value: the operator, the basis vectors, and the eigenvalues, $\hat{H}|j\rangle =e_{j}|j\rangle$, the expectation, $\mathrm{tr}\;[\hat{\rho }\hat{H}]=\bar{e}$, and its total value, $N\bar{e}=E$.^8^ The terms in parentheses in the utility representations are the marginal utilities in terms of the marginal values.

Because the Hamiltonian is conserved, so is value. This is the first law. Equation (2.14) is the second law ([11], p. 44).

## 3. Conclusion: resolving the Allais paradox

To conclude, we provide an example of how to use ensembles and quantum decision theory; we turn to the Allais paradox. Its keystone is the apparent violation of vNM/Savage independence [13]. In our formalism, we only assumed orthogonality for the basis vectors; each outcome being separate and distinct from every other outcome. Thus, the Allais paradox is resolved before we start with the analysis, but this neither shows how to use ensembles nor quantum theory.

In the quantum framework, the researcher’s objective, like the physicist's, is to create a model of the Hamiltonian that adequately explains the data. To do this, we use List & Haigh’s experiment on two cohorts of people, college students and professional traders, testing the Allais paradox [23]. The cohorts were consistent with each other when evaluating simple, binary choice games but not for the complex composite game.

Following Allais, List & Haigh presented each cohort with three different games:

(i) Choice A: win $7 with certainty. Choice B: win $7 with 75% probability, $10 with 20% probability, and $0 with 5% probability.
(ii) Choice A: win $7 with 25% probability and win $0 with 75% probability. Choice B: win $10 with 20% probability and win $0 with 80% probability.
(iii) Choose A or B between each of the previous two games. The payout will be from one of the two games, (i) or (ii), with a 50% probability.

We begin by constructing the Hamiltonians for each of the games. Assuming no quantum effects, the Hamiltonians are diagonal matrices of the expected payouts for each of the games,

3.1 $${\hat{H}}_{i}=\left[\begin{array}{cc} 7 & \\ & 7.25 \end{array}\right],\;{\hat{H}}_{ii}=\left[\begin{array}{cc} 1.75 & \\ & 2 \end{array}\right],\;\text{and}\;{\hat{H}}_{iii}=\frac{1}{2}\left[\begin{array}{cccc} 8.75 & & & \\ & 9 & & \\ & & 9 & \\ & & & 9.25 \end{array}\right].$$

The purely diagonal Hamiltonian means that each of the possible states has a uniform selection functional weight ([21], pp. 119–123) and is equivalent to an equal *a priori* probability of each state’s occupancy, vNM independence. The assumption of equal *a priori* probability maximizes the ensemble’s von Neumann entropy. While this is not always an accurate assumption, it provides an absolute reference point and should always be the first step of any analysis.

Next, we construct the empirical distributions with just the eigenvalues of the observations for the students,

3.2 $${\hat{\rho }}_{S,i}=\frac{1}{32}\left[\begin{array}{cc} 15 & \\ & 17 \end{array}\right],\;{\hat{\rho }}_{S,ii}=\frac{1}{32}\left[\begin{array}{cc} 8 & \\ & 24 \end{array}\right],\;\text{and}\;{\hat{\rho }}_{S,iii}=\frac{1}{30}\left[\begin{array}{cccc} 4 & & & \\ & 13 & & \\ & & 3 & \\ & & & 10 \end{array}\right]$$

and the traders,

3.3 $${\hat{\rho }}_{T,i}=\frac{1}{27}\left[\begin{array}{cc} 8 & \\ & 19 \end{array}\right],\;{\hat{\rho }}_{T,ii}=\frac{1}{27}\left[\begin{array}{cc} 3 & \\ & 24 \end{array}\right],\;\text{and}\;{\hat{\rho }}_{T,iii}=\frac{1}{54}\left[\begin{array}{cccc} 8 & & & \\ & 7 & & \\ & & 9 & \\ & & & 30 \end{array}\right].$$

We find that the differences between ${\hat{\rho }}_{S,iii}$ and ${\hat{\rho }}_{T,iii}$ are not due to paradox as List & Haigh suggest, but rather are due to the lack of experience of the students in assessing risk. The students’ strategy is ${\hat{\rho }}_{S,iii}={\hat{\rho }}_{S,i}⊗{\hat{\rho }}_{S,ii}$ with a *p*-value of 0.0021 strongly suggesting that they naively evaluated the joint game, (iii), as two independent games. Whereas the traders correctly assessed the joint game and followed the canonical distribution, equation (2.9) using ${\hat{H}}_{iii}$ with a* p*-value of 0.012.

Using statistical ensembles, we show that the observed results do not violate the vNM independence assumption. However, this is only part of the picture, as some anomalies in the observed distributions can only be explained in a quantum framework.

Looking more closely at the data, we see a clear pattern in the students and traders, where (i). A is preferred more than what is canonically represented. To understand why this is, we need to look at the temperatures of each cohort’s choices for each game.

Each cohort represents a distinct but uniform population; each game must have the same temperature for each cohort by the third law. The fact that they do not suggests that something is skewing their choices at the quantum level. The students had temperatures of $2.0 and $0.23 for games (i) and (ii), respectively. The traders had a similar pattern of $0.29, $0.12 and $0.23 for games (i), (ii) and (iii), respectively, suggesting that they are less risk-averse than the students, while retaining the ambiguity aversion [3], the preference for (i).A. We can modify the Hamiltonian by adjusting the off diagonal matrix elements to account for the observed quantum interferences [2], seeking to get a single coefficient that collapses the observed traders’ preferences to have a uniform temperature. We can follow a similar procedure for the students.

The stronger preference for the guaranteed return of choice (i).A is apparent for both cohorts. However, it results in a lower overall utility for both. While the traders could evaluate the joint game with a higher utility, being nearly canonical, their entanglement of a slight ambiguity aversion resulted in slightly lower utility/von Neumann entropy from the maximum possible.

## Data Availability

All of the data are contained in Allais Analysis.R and consists of the transposed data from [23]. The data are provided in the electronic supplementary material [24].

## Footnotes

1 All of the axioms presented here are adapted from Ballentine’s formulation of quantum mechanics [17].

2 We use Dirac notation to represent vectors, tensors and matrices.

4 This section is truly an amalgam of the four different approaches, even if they are not directly cited.

5 We include the Hamiltonian in this list along with all of the different commodities within the economy.

6 From this point forward $\hat{M}$ will be used exclusively to represent money and not be a generic observable.

7 The Hamiltonian’s ensemble measure in traditional thermodynamics is either E or U. Because of the word utility and its reference in economics as u, we use E for the ensemble’s value.

8 This proof follows the same approach as ([7], pp. 217–218), and is essentially a duplicate with only the notation changed.

## Appendix A. Proofs

Proof of theorem 2.3. Expand the time-dependent operator around an arbitrary time ϵ,

A 1 $$\hat{U}(\epsilon )=\hat{I}-i\frac{\hat{H}}{c}\epsilon +O(\epsilon^{2}).$$

Since $\hat{U}(t)$ is a unitary operator, ${\hat{U}}^{†}(t)\hat{U}(t)=\hat{I}$. This results in the new operator, $\hat{H}$, being Hermitian, ${\hat{H}}^{†}=\hat{H}$. Neglecting the higher order terms of equation (A 1),

$$\begin{array}{rl} |\psi \rangle \;(\epsilon +t) & \;=\left(\hat{I}-i\frac{\hat{H}}{c}\epsilon \right)|\psi \rangle \;(t),\\ |\psi \rangle \;(\epsilon +t)-|\psi \rangle \;(t) & \;=\left(\hat{I}-i\frac{\hat{H}}{c}\epsilon \right)|\psi \rangle \;(t)-|\psi \rangle \;(t),\\ i\;c\frac{|\psi \rangle \;(\epsilon +t)-|\psi \rangle \;(t)}{\epsilon } & \;=\hat{H}|\psi \rangle \;(t)\\ \mathrm{and}\;i\;c\lim_{\epsilon \to 0}\frac{|\psi \rangle \;(\epsilon +t)-|\psi \rangle \;(t)}{\epsilon } & \;=i\;c\frac{d}{dt}|\psi \rangle \;(t)=\hat{H}|\psi \rangle \;(t). \end{array}$$

Proof of lemma 2.10. See ([25], pp. 43–45).
Proof of theorem 2.11. Observing a density matrix, we have $|s_{j}\rangle =\hat{\rho }|j\rangle =\lambda_{j}|j\rangle$. By theorem 2.19, the subjective probability is equivalent to the eigenvalues of the density matrix, $s(\lambda_{j})=\lambda_{j}$. Thus, $\sum_{j}|s_{j}\rangle =s$.
Proof of therem 2.16. We define the Hilbert space of wager P as G with |ψ⟩∈G. There are two possible strategies associated with $\hat{\rho }|j\rangle =\lambda_{j}|j\rangle$. We associate |1⟩ if the event P obtains and |2⟩ if it does not. From the definition of the safety equivalent wager, the observable $\hat{M}$ shares a basis with $\hat{\rho }$. Thus, $\hat{M},\;\hat{M}|j\rangle =m_{j}|j\rangle$, where $\langle M\rangle =\lambda_{1}m_{1}+\lambda_{2}m_{2}$.

(a) *General properties* For $\hat{M}$, we can show the following:
  (i) Since $\lambda_{2}=1-\lambda_{1}$, $\lambda_{1}\in [0,1]$, and probability is continuous, $\hat{M}$ is intern.
  (ii) $\lambda_{1}=1∴\hat{M}=m_{1}$.
  (iii) From (i), since $\lambda_{1}$ is always positive, $\hat{M}$ is increasing in both $m_{1}$ and $m_{2}$.
  (iv) Trivial to show from (i).
  (v) $m_{1}\lambda_{1}+m_{2}(1-\lambda_{1})=m_{2}\lambda_{2}+m_{1}(1-\lambda_{2})$. Since $\lambda_{2}=1-\lambda_{1}$, $\hat{M}=\hat{M}$.
  (vi) Let $p_{j}$ and $q_{j}$ be the eigenvalues of the density matrices associated with the expectation of $\left\langle M_{P}\right\rangle$ and $\left\langle M_{Q}\right\rangle$, respectively.
    — If $\exists (m_{1},m_{2})\in R$ such that $p_{1}m_{1}+p_{2}m_{2}=q_{1}m_{1}+q_{2}m_{2}$, then $p_{j}=q_{j}\;∴|\psi_{P}\rangle =|\psi_{Q}\rangle$
    — The same holds $\forall (m_{1},m_{2})\in R$.
    — From the above two statements, if $|\psi_{P}\rangle =|\psi_{Q}\rangle ∴G_{P}∼G_{Q}.$
(b) The consequence of (a).(vi) is that indifference only exists if the strategy density matrices and the observables share the same orthonormal basis, the density matrices commuting, lemma 2.10.

Proof of theorem 2.17. We consider a game, $G_{PQ}=G_{P}⊗G_{Q}$, where $\{|13\rangle ,|14\rangle ,|23\rangle ,|24\rangle \}\in G_{PQ}$; and ${\hat{\rho }}_{PQ}|jk\rangle =\lambda_{jk}|jk\rangle$ is associated with $\hat{M},\;\hat{M}|jk\rangle =m_{jk}|jk\rangle$.

(i) $$\begin{array}{rl} & \left(m_{13}\frac{\lambda_{13}}{\lambda_{13}+\lambda_{14}}+m_{14}\frac{\lambda_{14}}{\lambda_{13}+\lambda_{14}}\right)(\lambda_{13}+\lambda_{14})\\ & \;\;\;+\left(m_{23}\frac{\lambda_{23}}{\lambda_{23}+\lambda_{24}}+m_{24}\frac{\lambda_{24}}{\lambda_{23}+\lambda_{24}}\right)(\lambda_{23}+\lambda_{24})\\ & \;\;=\left(m_{13}\frac{\lambda_{13}}{\lambda_{13}+\lambda_{23}}+m_{23}\frac{\lambda_{23}}{\lambda_{13}+\lambda_{23}}\right)(\lambda_{13}+\lambda_{23})\\ & \;\;\;+\left(m_{14}\frac{\lambda_{14}}{\lambda_{14}+\lambda_{24}}+m_{24}\frac{\lambda_{24}}{\lambda_{14}+\lambda_{24}}\right)(\lambda_{14}+\lambda_{24}) \end{array}$$
  $$\begin{array}{rl} & m_{13}\lambda_{13}+m_{14}\lambda_{14}+m_{23}\lambda_{23}+m_{24}\lambda_{24}\\ & \;\;=m_{13}\lambda_{13}+m_{23}\lambda_{23}+m_{14}\lambda_{14}+m_{24}\lambda_{24.} \end{array}$$
(ii) Given an observable, $\hat{M}$, that is degenerate in |14⟩,|23⟩ and |24⟩
  $$\begin{array}{rl} & \left(m_{13}\frac{\lambda_{13}}{\lambda_{13}+\lambda_{14}}+m_{14}\frac{\lambda_{14}}{\lambda_{13}+\lambda_{14}}\right)(\lambda_{13}+\lambda_{14})+m_{14}(\lambda_{23}+\lambda_{24})\\ & \;\;=m_{13}\lambda_{13}+m_{14}(\lambda_{14}+\lambda_{23}+\lambda_{24}). \end{array}$$

Proof of theorem 2.19. Consider two wagers, P and Q, their games $\{|1\rangle ,\;|2\rangle \}\in G_{P}$ and $\{|3\rangle ,\;|4\rangle \}\in G_{Q}$, and their joint game, $G_{PQ}=G_{P}⊗G_{Q}$, where $\{|13\rangle ,|14\rangle ,|23\rangle ,|24\rangle \}\in G_{PQ}$. And ${\hat{\rho }}_{PQ}|jk\rangle =\lambda_{jk}|jk\rangle$ is associated with $\hat{M},\;\hat{M}|jk\rangle =m_{jk}|jk\rangle$. Where

— For $G_{P}\;|1\rangle :\;\lambda_{1}=\lambda_{13}+\lambda_{14},\;|2\rangle :\;\lambda_{2}=\lambda_{23}+\lambda_{24}$
— $m_{1}\lambda_{1}=m_{13}\lambda_{13}+m_{14}\lambda_{14},\;m_{2}\lambda_{2}=m_{23}\lambda_{23}+m_{24}\lambda_{24}$
— For $G_{Q}\;|3\rangle :\;\lambda_{3}=\lambda_{13}+\lambda_{23},\;|4\rangle :\;\lambda_{4}=\lambda_{14}+\lambda_{24}$
— $m_{3}\lambda_{3}=m_{13}\lambda_{13}+m_{23}\lambda_{23},\;m_{4}\lambda_{4}=m_{14}\lambda_{14}+m_{24}\lambda_{24}$.

Since $\lambda_{2}=1-\lambda_{1}$, we can write,^8^

$$\begin{array}{rl} & s(\lambda_{1})U(m_{1})+(1-s(\lambda_{1}))U(m_{2})\\ & \;\;=s(1-\lambda_{1})U(m_{2})+(1-s(1-\lambda_{1}))U(m_{1}). \end{array}$$

Hence, $1-s(\lambda_{1})=s(1-\lambda_{1}).$ From theorem 2.16,

$$\begin{array}{rl} & s(\lambda_{13})U(m_{13})+(1-s(\lambda_{13}))U(m_{2})=s(\lambda_{3})U(m_{1})+(1-s(\lambda_{3}))U(m_{2})\\ & \;=s(\lambda_{1})\left[s\left(\frac{\lambda_{13}}{\lambda_{1}}\right)U(m_{13})+\left(1-s\left(\frac{\lambda_{13}}{\lambda_{1}}\right)\right)U(m_{2})\right]+(1-s(\lambda_{3}))U(m_{2})\\ & \;=s(\lambda_{1})[s(\lambda_{3})U(m_{13})+(1-s(\lambda_{3}))U(m_{2})]+(1-s(\lambda_{3}))U(m_{2}). \end{array}$$

Hence,

A 2 $$s(\lambda_{13})=s(\lambda_{1})s(\lambda_{3}).$$

If we take $\lambda_{1}<\lambda_{3}$ and $m_{13}>m_{2}$,

$$s(\lambda_{1})U(m_{13})+(1-s(\lambda_{1}))U(m_{2})<s(\lambda_{3})U(m_{13})+(1-s(\lambda_{3}))U(m_{2})$$

and thus

$$s(\lambda_{1})(U(m_{13})-U(m_{2}))<s(\lambda_{3})(U(m_{13})-U(m_{2}));$$

by the monotony of U, $s(\lambda_{1})<s(\lambda_{3})$, equation (A 2) implies $s(\lambda_{j})=\lambda_{j}$.
Proof of theorem 2.25.

(i) If the Hamiltonian of the ensemble is bounded, $\mathrm{tr}\;[{\text{e}}^{-\beta \hat{H}}]<\infty$, then entropy is a continuous functional.
(ii) theorem 2.16 proved relation (a) for any quantum observable.
(iii) theorem 2.17 proved relation (b) for any quantum observable.
(iv) Since entropy is homogeneous of the first degree, it is unique up to a linear transformation.
(v) Since log⁡q in equation (2.10) is a functional of the $\beta_{k}$ and not of the ${\bar{x}}_{k}$ it is an additive constant to $\bar{s}$.
(vi) It is not necessarily the case for the $X_{k}$s and N that entropy is an increasing functional. In the case when the expenditure of $X_{k}$ is minimized, $\partial S/\partial X_{k}>0$. If $X_{k}$ does not affect entropy, $\partial S/\partial X_{k}=0$. And in the case of the maximization of the expenditure of $X_{k}$, $\partial S/\partial X_{k}<0$. We see a similar occurrence with N, but where when the population density is low for a given $\left\{X_{k}\right\}$, log⁡q>0, and entropy is an increasing functional of N. When the population density is high, log⁡q<0, and entropy is a decreasing functional of N. When log⁡q=0, $\bar{s}$ is extensive in the ${\bar{x}}_{k}$ and is scale independent.
  The strength of preference is represented by $\partial S/\partial X_{k}$. The more positive, the more it is wanted. The more negative, the more it is reviled. And when it is zero, it has no effect on entropy and we are indifferent to it.
  We can see that Pfanzagl’s requirement that utility be an increasing functional of a commodity did not fully express the possible conditions of utility and that he implicitly assumed that the commodity was a ‘good’ and not a ‘bad’.
